# Supplementary material for: Are carbon emissions trading and green financial instruments synergistic? -Comprehensive quantitative research based on content analysis
Source: PLoS One. 2024 Mar 7;19(3):e0298601. doi: 10.1371/journal.pone.0298601 (PMC10919601; doi:10.1371/journal.pone.0298601)
Supplement: S1 Data — (ZIP) [file pone.0298601.s001.zip › Data/地级市Z_F工作报告.zip!/╣π╬≈/│τ╫≤╩╨/└┤╫╘╣1⁄2╓┌║┼í╛┬φ┐╦╩2╛▌═°í┐_2017.docx]

各位代表：

现在，我代表市人民政府，向大会报告政府工作，请予审议，并请市政协委员和其他列席会议的同志提出意见。

一、 2016年工作回顾

2016年是“十三五”开局之年，也是全面建成小康社会决胜阶段的开局之年。一年来，面对错综复杂的发展环境，我们在自治区党委、政府和市委的坚强领导下，以“两学一做”学习教育为动力，牢固树立新发展理念，扎实做好“两篇大文章”，打好“四大攻坚战”，经济社会发展总体平稳，经济呈现缓中趋稳、稳中向好的发展态势，民生持续改善，社会和谐稳定，实现了“十三五”良好开局。初步预计，全市地区生产总值738亿元，同比增长7.8%；财政收入58.2亿元，完成全年预算任务；规模以上工业总产值748.98亿元，增长14.0%；规模以上工业增加值250亿元，增长7.2%；固定资产投资830亿元，增长20.0%，增速位居全区第二；外贸进出口总额1269.9亿元人民币，占全区总量比重达39.6%，同比提高0.2个百分点；社会消费品零售总额131.3亿元，增长10.1%；城镇居民人均可支配收入26555元，增长7.8%，增速位居全区前列；农村居民人均可支配收入9810元，增长10.0%，较好完成了全年经济社会发展各项目标任务，向与全国全区同步全面建成小康社会目标迈出了坚实的一步。

一年来，我们主要做了以下工作：

（一）综合施策促发展，经济保持稳定增长。面对经济下行压力加大的态势，我们始终保持坚定的战略定力，全面贯彻落实自治区41条降低实体经济企业成本措施，努力把握新机遇、寻找新动力、培育新增长点，制定崇左市21条稳增长降成本措施，出台口岸经济大发展等6个三年行动计划，综合施策，精准发力，以政策集成效应推动发展。完成一般公共预算支出204.88亿元，增长10.7%，盘活财政存量资金41.18亿元，财政资金使用效率明显提高。设立旅游发展基金、城市发展基金，撬动30亿元社会资本参与建设，有力推动了经济社会发展。

（二）深化改革激活力，发展动力明显增强。深入推进供给侧结构性改革，加快“三去一降一补”，取得初步成效。加快去产能步伐，妥善化解扶绥新帝矿业煤炭过剩产能。出台增加购房补贴等房地产去库存政策，全市商品房实际销售面积增长12.2%；推进棚户区改造货币化安置，完成安置4458套。积极稳妥防风险去杠杆，成功对左江永凯、凭祥才源等制糖企业并购重组。千方百计降成本，落实减费降税政策，企业税费负担减少3.56亿元；推动15家企业纳入电力直接交易范围，降低成本1.33亿元，锰企业产能利用率由年初的22.3%提高到41.8%。加大力度补短板，全市财政用于民生领域支出166.9亿元，增长14.3%，比一般公共预算支出增幅高3.6个百分点；民生支出占一般公共支出比重达81.5%，同比提高2.6个百分点。深化行政审批制度改革，率先在全区实施市县乡三级权力清单和责任清单“两单合一”工作，实现权责一致，开创了“权责对等”的先河；“先照后证”等商事制度改革深入实施，全市新增注册资本增长101.7%。中泰产业园体制改革不断深化，授予县级行政审批、财政投资管理等职能。加快沿边金融综合改革试验区建设，推动建设边民互市贸易结算中心，跨境人民币结算量保持全区第一。“一线放开，二线管住”跨境劳务合作模式试点实施。全面开展农村土地确权工作，全市可发证率达69.4%。供销合作等重点领域改革顺利推进。

（三）扩大开放促合作，开放型经济水平加速提升。围绕做好口岸经济发展大文章，积极参与“一带一路”建设，主动融入“双核驱动、三区统筹”战略，开放合作不断取得新成效。开放合作新平台加快搭建，凭祥重点开发开放试验区获批设立，中国—东盟边境贸易凭祥（卡凤）国检试验区成为全国首个国检试验区，凭祥综合保税区二期启动建设。对外贸易保持领先，外贸进出口总额连续8年稳居全区第一，边境小额贸易进出口总额占全区八成左右。边境加工业快速发展，凭祥—宁明贸易加工区、龙州水口边贸加工扶贫产业园加快建设，新增7家规模以上口岸加工企业，口岸加工业产值30.5亿元，增长54.8%。先行先试取得新进展，“广贸通”平台上线运行，友谊关口岸“单一窗口”改革、边境小额贸易出口“简单申报”工作加快推进。互联互通水平实现新提升，边境贸易货物监管中心实现试运营，友谊关—友谊国际口岸货运专用通道建成使用，友谊关口岸获批开展签证业务，水口口岸升格为国际性口岸。区域合作不断深化，与28个部门签订合作协议，与越南高平省等签署4个谅解备忘录，发展空间进一步拓展。

（四）打造品牌筑优势，文化旅游提速发展。围绕做好文化旅游发展大文章，大力实施“发现山水崇左·圆梦别样桂林”文化旅游发展战略，推动文化旅游产业和绿色生态提速发展。文化旅游品牌不断打响，左江花山岩画文化景观成功列为世界文化遗产，壮族文化走向世界；凭祥市列入首批国家全域旅游示范区创建名单，大新县成功创建广西特色旅游名县；中越德天·板约国际旅游合作区建设加快推进，德天跨国瀑布景区列入创建国家5A级景区预备名单，宁明花山景区、崇左石景林·园博园、大新德天·老木棉景区获批为4A级景区。重大旅游项目加快建设，扶绥龙谷湾旅游休闲度假区竣工试业，凭祥红木文博城等项目建设有序推进。预计全市累计接待游客2030万人次，增长27.1%；实现旅游总消费182亿元，增长37.5%。生态建设和环境保护实现新提升，主要江河地表水和集中式饮用水水质达标率均达100%，空气质量综合排名全区第三，荣获“国家森林城市”称号。

（五）调优结构促转型，新旧动能加快转换。围绕打好产业转型升级攻坚战，大力实施创新驱动发展战略，促进产业提质增效。启动工业园区标准厂房建设，建成19万平方米，园区综合承载能力进一步提升，全市工业园区实现工业总产值535亿元，增长13.3%。传统产业加快转型升级，糖业产业链不断延伸，好青春甘蔗醋饮料、金冠糖果等项目建成投产，湘桂活性干酵母等项目加快推进，预计全年糖业产值增长15.0%。矿业逐步向高端化发展，中铝国盛稀土冶炼分离项目成为国家智能制造试点示范项目，南国铜业等项目加快建设，广西大锰锂离子动力电池等项目开工建设，预计全年锰业产值增长4.2%。新兴产业加快发展，龙赞林业循环经济产业园一期3个项目竣工投产，金梧桐果品加工基地、金亚新能源电动车等项目开工建设。现代农业创新发展，全年粮食总产量51.9万吨；落实“双高”基地建设面积32.42万亩，“四化”规模进一步扩大，平均亩产达6吨以上；建设区市县乡四级现代特色农业（核心）示范区85个，新增自治区认定示范区27个；新增农民合作社897个、家庭农场70个。现代服务业提速发展，中国边贸第一城、市农贸市场等项目竣工运营，跨境电子商务快速发展。

（六）狠抓项目扩投资，发展后劲不断增强。围绕打好基础设施建设攻坚战，集中力量推进一批重大项目建设，固定资产投资增速持续位居全区前列。落实2亿元工作经费，推动项目前期工作顺利开展。133个市级层面以上统筹推进重大项目完成投资132.4亿元，其中35个自治区层面重大项目完成投资56.9亿元。崇左至靖西高速公路建成通车，崇左港将军岭作业区试运行，濑湍作业区建设步伐加快，驮英水库及灌区工程开工建设。招商引资工作有效推进，预计全市区外招商引资项目到位资金295亿元，全口径实际利用外资1.69亿美元。

（七）统筹城乡共发展，新型城镇化加快推进。围绕打好新型城镇化攻坚战，加快建设山水园林城市，中心城区建成区面积扩大到33平方公里，预计全市城镇化率提高到37.08%。全面实施中心城市提升完善工程，城区水系改造提升、路网桥梁、文化教育、园林绿化、商贸及专业市场、公共服务设施、房地产7大系列项目加快推进，东盟大道等项目建成通车，市体育中心、崇左大桥等项目加快建设。保障性安居工程建设进展顺利，新开工棚户区改造11841套，基本建成5302套，完成农村危房改造11200户。实施大县城战略，凭祥国家中小城市综合改革试点有序推进。培育发展特色城镇，新和镇、雷平镇、山圩镇、水口镇等名镇、百镇建设示范工程加快建设。深入推进“美丽崇左·生态乡村”建设，成功创建28个自治区级“绿色村屯”。城乡路网不断完善，隆安经进结至天等、德保至天等二级公路建成通车，100%行政村通沥青（水泥）路。

（八）精准发力促扶贫，脱贫攻坚首战告捷。围绕打好农村全面脱贫攻坚战，组建9个脱贫攻坚专责小组，出台脱贫攻坚“十大行动” “八个一批”精准帮扶方案以及23个行动方案。落实"4321"干部精准帮扶机制，开展“百企联百村”“万人帮万户”活动，建立“一户一册一卡”精准帮扶台账，实现帮扶对象全覆盖。整合涉农资金11.54亿元，实施1.15万个脱贫攻坚项目，进一步夯实脱贫基础。大力发展“种养贸游工”五大产业扶贫，边贸扶贫经验得到中央媒体宣传报道。全年累计发放扶贫小额贷款15.82亿元，惠及3.22万户贫困户。有序推进易地扶贫搬迁，全市33个安置点全部开工建设，竣工住房2174套。社会兜底、教育扶贫、医疗扶贫等统筹推进，全年2.14万户9万贫困人口脱贫、47个贫困村脱贫摘帽，全面完成自治区下达的年度任务。

（九）保障民生增福祉，社会大局和谐稳定。全面完成为民办实事工程项目。教育“双百工程”加快推进，凭祥市义务教育均衡发展经验在全国推广，天等、大新、宁明通过自治区义务教育均衡发展督导评估，广西民族师范学院附属中学通过自治区示范性普通高中评估验收；全市学前三年毛入园率达85.6%，提前达到2020年全国目标值，义务教育巩固率提高4.2个百分点，高中阶段毛入学率提高1.8个百分点，市本级高考一本上线率提高4.8个百分点；广西城市职业学院崇左校区开工建设。科技创新能力日益增强，每万人发明专利拥有量增长83.2%，科技成果转化8项。文化事业繁荣发展，成功举办2016年广西“壮族三月三”民族体育欢乐节暨崇左花山文化节、本色花山·大地飞歌晚会。卫生计生事业稳步推进，“全面两孩”政策稳妥实施，新农合参合率99.69%，率先在全区实施农村贫困人口新农合住院补偿和大病保险倾斜政策。就业保持稳定，城镇新增就业1.53万人，城镇登记失业率2.9%；发放农民工创业担保贷款5173万元，农村劳动力新增转移就业3.53万人。社会保障水平稳步提高，全市城乡居民基本养老保险参保率97.9%，城市居民最低生活保障标准提高到430元/月，农村居民最低生活保障标准提高到3200元/年。物价水平保持稳定，预计全市CPI涨幅1.8%。社会保持和谐稳定，率先建成全区首个边境综治维稳信息中心，边境管控、社会治安综合治理、安全生产、应急管理、矛盾纠纷化解等工作持续加强，维护社会稳定动态八项工作综合总分、群众安全感持续排名全区前列，荣获“全国法治宣传教育先进城市”称号。双拥工作实现历史性突破，荣获“全国双拥模范城”称号，龙州再次荣获“全国双拥模范县”称号。

（十）提升效能塑形象，政府建设不断加强。扎实开展“两学一做”学习教育，政府效能和执行力全面提升。加快政府职能转变和机构改革，全面完成事业单位分类工作。推行“互联网+税务”模式，实现92.6%的纳税人通过互联网进行税务申报。强化非公人士反映问题“直通车”制度，解决了一批企业热点难点问题。严格规范行政执法行为，依法行政能力和法治政府建设不断增强。自觉接受人大和政协监督，人大代表建议、政协委员提案办结率100%。政府预算、部门预算和“三公”经费预算全部公开。扎实推进公务用车制度改革。狠抓全面从严治党，切实加强廉政建设，持续推进作风转变，着力提高服务水平。充分发挥行政监察职能，依法进行审计监督，坚持有腐必反、有贪必肃，营造风清气正的良好环境。

一年来，我市人力资源、统计调查、水库移民、国有资产监管、食品药品监管、质量技术监督、新闻出版、广播影视、侨务、二轻、宗教、涉台事务、机关事务管理、接待、驻邕管理、驻京联络、地震、人防、边防、税务、银监、供电、无线电管理、气象、水文、消防、海关、海事、档案、保密、国家安全、老龄、地方志等工作取得新进展。银行、保险、邮政、通信、石油、烟草、盐业等行业和工会、共青团、妇联、工商联、科协、文联、侨联、残联、社科联、红十字会等群团组织在经济社会发展中发挥了重要作用，作出了积极贡献。

各位代表！过去的一年，在严峻复杂的经济形势下，我们凝心聚力、砥砺奋进，顶住了压力，经受了考验，增速不断提高，发展质量和效益持续向好，为我市加快发展、赶超跨越打下了坚实基础。这是市委统揽全局、科学谋划的结果，是市人大、市政协有效监督、大力支持的结果，是全市广大干部群众众志成城、合力攻坚的结果，是离退休干部建言献策、鼓励支持的结果，是驻崇左人民解放军、武警部队和社会各界积极参与、鼎力支持的结果。在此，我谨代表市人民政府，向全市各族人民，向人大代表、政协委员，向各民主党派、工商联、无党派人士、人民团体，向驻崇左人民解放军、武警部队官兵，向所有关心支持崇左发展的社会各界，表示衷心的感谢和崇高的敬意！

在肯定成绩的同时，我们也清醒看到，去年我市部分经济指标增幅出现回落，暴露了经济社会发展中的深层次矛盾和问题。主要表现在：经济总量仍然偏小，重大项目还不够多，对外贸易形势严峻，财税增收困难，经济基础还较为薄弱；产业结构不够合理，科技创新能力不足，转型升级步伐不快，新产业处于培育阶段，抗市场风险能力较弱；公共服务水平与人民期盼还有差距，贫困程度较深，脱贫攻坚任务艰巨；政府自身建设仍需加强，干部作风尚需加快转变等。对此，我们必须高度重视，采取有效措施，认真加以解决。

二、 2017年工作安排

2017年是实施“十三五”规划的重要一年，也是我市做好“两篇大文章”、打好“四大攻坚战”的第二个攻坚年。从宏观上看，国内外经济形势依然错综复杂，结构调整的压力依然较大，我市面临着加快发展和加快转型的双重任务，随着国家深入实施“一带一路”战略，自治区加快构建“四维支撑”“四沿联动”开放发展新格局，我市区位优势进一步凸显，仍处于加快发展的战略机遇期。从微观上看，糖锰价格处于较高水平、工业园区建设全面加速、凭祥重点开发开放试验区加快建设、花山岩画申遗成功后旅游业快速发展，积极因素不断涌现。压力与希望共生，挑战与机遇并存，我们必须抢抓机遇，勇迎挑战，将我市经济社会发展推向质量更高、效益更好、更可持续的新阶段。

今年政府工作的总体要求是：全面贯彻党的十八大、十八届三中、四中、五中、六中全会和中央、全区、全市经济工作会议精神，深入学习贯彻习近平总书记系列重要讲话精神，按照统筹推进“五位一体”总体布局和协调推进“四个全面”战略布局的要求，坚持稳中求进工作总基调，牢固树立和贯彻落实新发展理念，适应把握引领经济发展新常态，坚持以提高发展质量和效益为中心，以推进供给侧结构性改革为主线，围绕市第四次党代会重大部署，保持战略定力，继续做好“两篇大文章”、打好“四大攻坚战”，扎实推进稳增长、促改革、调结构、惠民生、防风险各项工作，促进经济社会平稳健康发展，为与全国全区同步全面建成小康社会打下坚实基础，以优异成绩迎接党的十九大胜利召开。

今年经济社会发展主要预期目标是：地区生产总值增长8%左右，财政收入增长3%左右，规模以上工业增加值增长8%，固定资产投资增长15%，社会消费品零售总额增长10%，外贸进出口总额增长10%，城乡居民人均可支配收入实际增长8%以上，节能减排控制在自治区下达目标内。

制定上述目标，主要是充分考虑了我市当前发展形势及经济运行等综合因素，着眼于增强发展动力、厚植发展优势、补齐发展短板，致力于调结构、提质量、惠民生、利长远。为实现上述目标，我们将围绕做好“两篇大文章”、打好“四大攻坚战”的战略任务，重点抓好以下八个方面的工作。

（一）坚持深化改革，不断增强经济社会发展动力。

深化供给侧结构性改革。继续抓好“三去一降一补”任务落实，不断提高全要素生产率。去产能方面，加快淘汰一批落后产能，积极推进宁明丰浩、大新世纪飞龙、天等东糖俊杰等制糖企业兼并重组工作，推动锰矿资源集中到综合实力强的大型企业。去库存方面，推进棚户区改造货币化安置，力争全年棚改货币化安置比例达70%以上；落实房地产去库存优惠政策，加大财政补贴力度，鼓励吸引农民工等新市民进城购房。去杠杆方面，建立健全政府性债务预算管理制度，积极盘活存量资产；推动企业股权融资、金融租赁等，扩大直接融资渠道；加大金融风险管控力度，妥善处置银行不良贷款。降成本方面，积极参与售电侧改革，扩大工业企业参与电力直接交易范围，降低企业用电成本。补短板方面，加强基础设施薄弱环节和生态环境保护，着力提升人力资本水平，持续增强公共服务能力，夯实发展基础。

深化行政审批制度改革。编制并公布行政审批中介服务事项清单和公共服务事项目录清单，建立宽准入、严监管的管理模式。推进政府权责清单动态管理，规范行政权力透明运行。深化“先照后证”“六证合一、一照一码”“两证整合”等商事制度改革，积极推进“证照分离”改革。

深化财税体制改革。启动新一轮税收征管体制改革。建立科学规范的财政管理体制。完善政府预算体系，加强预算执行、预算绩效和政府债务管理。盘活用好财政存量资金，集中有限的资金用于稳增长、调结构、惠民生的重点领域和关键环节。

深化投融资体制改革。深入推进沿边金融综合改革，探索规范统一的边贸结算模式，拓宽本外币兑换特许业务范围，进一步扩大跨境人民币结算量，加快将凭祥打造为全区跨境贸易结算平台次中心。支持域外金融机构在崇左设立分支机构。大力推广政府购买公共服务、PPP等模式，创新政府和社会资本合作机制。进一步扩大债券市场融资，发行20亿元城投企业债券。积极发挥崇左市投资引导基金杠杆作用，加快与兴业银行等合作设立专项基金，探索设立中泰产业园产业发展基金。推动崇左市小微企业融资担保公司全面开展业务，缓解小微企业融资难题。

深入推进农业供给侧结构性改革。优化产品结构、经营结构和区域结构，调好调顺调优农业结构，规划建设甘蔗生产保护区。狠抓农产品标准化生产、品牌创建和质量安全监管，打造更多的绿色优质农产品。大力推进“互联网+现代农业”，加强农产品电子商务平台建设，推动农产品生产由生产导向逐步向消费导向转变，促进农业全产业链改造升级。加快推进农村土地承包经营权确权登记颁证工作，争取年内完成可发证率达总任务的95%以上。

深化相关体制机制改革。加快推进凭祥综合保税区、中泰产业园、凭祥边境经济合作区等园区体制改革，授予园区更多自主权。统筹推进市属国有平台公司、供销合作、城市综合执法等其他领域改革，支持司法体制、人才体制机制改革。

大力支持非公经济发展。加大创新服务非公经济工作机制，落实非公经济发展政策措施，以更加高效优质的服务，着力为非公经济企业解决发展难题。

（二）坚持开放发展，做大做强口岸经济。

加快推动优惠政策落地。加强对国家政策的研究，争取更多先行先试政策。推动自治区尽快出台《加快推进广西凭祥重点开发开放试验区的若干政策》，推动尽快设立试验区管委会，加快试验区建设总规和各专项规划编制，完善试验区重大项目库和储备库。积极争取龙州边境经济合作区获得国家批复，加快推动中越凭祥-同登跨境经济合作区、中国-东盟边境贸易龙州国检试验区申报建设，推动中泰产业园上升到两国政府层面共同建设，确保国家赋予的各项政策真正落地生效，为崇左开放发展探索新路子。

加快实施“口岸+”。继续实施加工贸易倍增计划，推动发展“口岸+加工制造业”。加快凭祥综合保税区二期建设，大力发展“保税+智造、物流、贸易、服务”。加快推进凭祥-宁明贸易加工区、龙州水口边贸加工扶贫产业园、凭祥红木文化创意产业园等园区建设。推动民之天食品厂、江南食品厂等企业扩大生产，推动金梧桐果品加工、越香园乳制品等项目竣工投产，力争全年规模以上企业口岸加工业产值达55亿元。大力发展“口岸+商贸物流”，加快建设中国-东盟（凭祥）农副产品物流中心、中越边境中药材商贸物流中心等项目。大力发展“口岸+特色城镇”，加快硕龙、友谊、水口、爱店等边境口岸城镇建设，加快构建口岸经济新业态。

优化口岸经济发展环境。加强口岸建设，争取水口口岸升格通过国家验收、友谊关口岸扩大开放获得国家批复，争取爱店、平而、岩应等升级为互市区。推进浦寨、弄怀一体化建设。创新口岸通关监管模式，推行“单一窗口”“两国一检”“一站式”“预约式”等通关模式，精简边境小额贸易出口“简单申报”手续，推动“市场采购”贸易方式扩大运用范围，提升通关效率。

强化招商引资。深化国际友城交流合作，努力开辟国际新市场。加强区域合作，承接产业转移。落实招商引资“一把手”工程，强化精准招商、务实招商理念，加强与全国知名商协会沟通联系，大力实施驻点招商、专业招商、以商招商、委托招商等，全力推动项目落地，力争全年区外招商引资项目到位资金303亿元以上。

（三）坚持绿色引领，推动文化旅游提档升级。

加强生态建设与保护。落实环境保护“党政同责”和“一岗双责”责任制，严格执行“大气十条”“水十条”“土十条”，落实“河长制”，强力整改中央环境保护督察反馈问题。大力推进节能减排，进一步控制主要污染物排放。推动全市各工业集聚区污水集中处理设施全部建成使用。加大生态建设力度，继续做好植树造林和森林抚育等工作，力争森林覆盖率提高到54.75%。巩固提升国家森林城市创建成果，推动龙峡山森林公园晋升国家级森林公园，力争新增2处湿地公园，营造山清水秀的自然生态。

提升文化旅游品位。加强花山岩画保护和利用，深入挖掘花山文化，推进花山题材艺术创作等工作，提升花山岩画景区文化品位，不断深化花山文化与花山旅游的融合。加快推进花山岩画博物馆、周氏兄弟现代艺术中心项目前期工作，推动左江花山音画夜游和大型实景演出项目年内实现运营，将花山文化旅游打造成为中国乃至世界的知名品牌。推进凭祥市国家全域旅游示范区、广西特色旅游名县名镇和A级景区、星级饭店创建工作，力争龙州县成功创建广西特色旅游名县，推动德天跨国瀑布景区成功创建5A级景区，新增4A级旅游景区5家以上、三星级以上旅游饭店6家，打造更多特色旅游品牌。

加快旅游项目建设。充分发挥左江花山投资集团公司的平台作用，做好旅游项目的规划设计及开发经营。加快推进中越德天·板约国际旅游合作区建设，加快一批重大基础设施项目建设，尽早实现跨境旅游。抓好花山岩画文化景区、明仕国家旅游度假区等重大旅游项目建设。推进德天至花山旅游大通道、南友高速公路花山互通等旅游道路建设。新建20座旅游厕所，完成27处旅游交通标识牌安装，加快打通服务游客“最后一公里”。

强化旅游宣传营销。创新旅游营销模式，拓宽旅游电商营销渠道。加强与国内重点区域旅游目的地和客源地的联动，扩大与国际旅游组织的交流合作。加快推进智慧旅游系统建设，实现全市主要景区和游客集散区域免费无线宽带全覆盖。力争全年接待游客2400万人次以上，实现旅游总消费230亿元以上。

（四）坚持创新发展，不断提升产业发展质量效益。

突出创新驱动。加快崇左高新技术产业开发区、中科院上海高等研究院（崇左）蔗糖检测分中心筹建工作。加强创新平台建设，加快崇左市中南大学联合研究中心建设，与中铝公司合建新材料研究院，与广西大锰合建新能源研究院。突出企业创新主体地位，引导企业进行技术、产品、业态等方面创新，力争在糖业、锰业、新材料、生态环保等重点领域的关键技术取得新突破。深入实施质量兴市战略，大力推动品牌和标准化建设。

推动园区提质发展。进一步完善园区规划，加快推进中泰产业园、扶绥空港经济区、中国-东盟青年产业园、凭祥边境经济合作区等园区建设。强力推进园区道路、给排水、供电、污水处理等基础设施建设，年内建成30万平方米标准厂房，企业入驻率达30%以上。

推动传统优势产业转型升级。深入实施糖业“二次创业”，大力发展糖深加工和糖业循环经济，加快湘桂-乐思福酵母及酵母抽提物等项目建设，推动崇左东亚糖业整体技改搬迁循环经济项目、中粮屯河风味糖浆、崇左食糖仓储物流中心等项目开工建设。大力推动矿业发展，落实扶持优惠政策，加快龙州氧化铝项目前期工作，推动新振锰业电解金属锰二期、中低碳锰铁和中信大锰布东锰深加工系列项目等开工建设；加快南国铜业项目建设，力争年底前一期项目竣工试产、二期项目开工建设。进一步增强建材企业竞争力。

加快培育新兴产业。加快推进新能源产业园的布局和项目建设，推动金亚新能源电动车、中信大锰三元正极材料、广西大锰锂离子动力电池项目竣工投产。推动稀土催化剂、稀土金属、稀土铝合金、稀土医疗材料等项目开工建设。大力发展林产业，推动龙赞林业循环经济产业园一期8个项目全部竣工投产。

大力发展现代服务业。加快现代服务业集聚区建设，推动中泰产业园泰国风情特色小镇、金凤湖畔商业街区、崇左市汽车城等项目开工建设。落实“电商广西、电商东盟”工程，积极发展跨境电子商务，推进中国-东盟（凭祥）电商产业园、龙州水口东盟国际商贸城等项目建设。培育发展大健康产业，加快中国（扶绥）乐养城、大新明仕养生养老生态园等项目建设，争取万达集团、泛海集团医养城项目落户崇左。

加快发展现代农业。落实粮食生产行政首长责任制，狠抓粮食生产，确保粮食播种面积稳定在180万亩、粮食总产量50万吨以上。坚定不移抓好甘蔗生产，咬定400万亩种植面积不放松，完成40万亩“双高”基地建设任务。加快现代特色农业（核心）示范区建设，年内新增自治区认定15个。大力发展“菜篮子”工程、秋冬种农业开发、富硒农业、生态循环农业和休闲农业，加快建设绿色生态畜禽和水产品基地。积极培育新型农业经营主体，年内新增农民合作社200家以上、家庭农场70家以上、牛羊养殖小区400家以上。继续抓好第一批4万亩耕地提质改造“旱改水”项目工作，力争上半年通过验收。

（五）坚持项目带动，不断夯实发展基础。

着力扩大项目投资。大力实施左右江革命老区重大工程建设三年行动计划，推进老区振兴发展。围绕产业结构调整、基础设施建设、民生改善等领域，统筹推进164个重大项目建设，力争年内完成投资 225亿元。强化项目前期工作，全市安排工作经费2.1亿元以上。引导扩大民间投资规模，保持投资高速增长态势。

加快现代交通体系建设。力争南宁至崇左城际铁路年内开工建设，加快推进崇左至凭祥高速铁路项目前期工作。推动隆安至硕龙高速公路、中越水口-驮隆界河二桥、渠黎至中泰产业园一级公路和龙州至凭祥、天等至仙桥二级公路等项目开工建设，推进崇左至水口高速公路、南宁新江经吴圩至扶绥一级公路、凭祥-宁明边贸加工区工业大道等项目建设，确保驮卢至大新二级公路等项目竣工通车。大力实施农村公路安全防护工程，进一步提升农村公路建设水平。

加强水利水运基础设施建设。加快渠珠水利枢纽工程、左江龙州至崇左Ⅲ级航道工程等项目前期工作。加快推进驮英水库及灌区工程项目建设，力争完成水库进场道路建设和移民安置等工作。大力实施农田水利设施、抗旱规划水源工程、农村饮水安全巩固提升等民生水利工程，不断夯实水利基础。

加快能源基础设施建设。大力实施县县通天燃气工程，力争年内开工建设崇左供气站。推动110千伏石林送变电工程、中泰产业园中电国际综合能源一期项目等建成使用，力争扶绥琦泉生物质热电联产、天等牛头岭风电场、江州区50兆瓦农光互补等项目年内实现并网发电。加快实施新一轮农村电网改造工程，提高农网供电能力和供电可靠性。

（六）坚持精准扶贫，加快脱贫攻坚步伐。

强化帮扶措施。进一步健全精准扶贫工作机制，调动各方力量参与脱贫攻坚。完善脱贫攻坚大数据管理平台，做好扶贫信息管理。加强农村低保和扶贫政策衔接，落实政策性保障兜底。建立健全脱贫攻坚督查、考核及责任追究制度，杜绝“数据脱贫”“被脱贫” “假脱贫”现象发生。全力做好电商扶贫、金融扶贫、教育扶贫、健康扶贫、社会扶贫等工作，全方位、多层面保障脱贫攻坚成效。年内完成11.1万贫困人口脱贫、88个贫困村和龙州、大新2个贫困县摘帽任务。

夯实贫困地区发展基础。加快推进农村屯级道路、人饮工程、危房改造、村级公共服务设施等一批基础设施项目建设。重点建设420个屯级道路项目，改造5900户贫困户危旧房，建设39 个贫困村公共服务中心，加快提升广播电视和宽带网络覆盖率，进一步补齐贫困地区发展短板。

突出抓好产业扶贫。围绕“种养贸游工”产业扶贫五大重点工作，加快实施一批“短、平、快”产业项目，增强贫困户脱贫发展后劲。抓好种植业扶贫，引导贫困户发展优质水果、中草药、食用菌等特色农产品。抓好养殖业扶贫，大力发展牛、羊等食草动物养殖，引导贫困户入股参与养殖小区建设。抓好边贸扶贫，争取国家批复实施边民互市贸易改革试点方案，引导更多贫困边民参与边境贸易或边贸加工，力争1.6万名贫困边民实现脱贫。抓好旅游扶贫，鼓励引导贫困群众发展乡村旅游，从旅游业发展中获得稳定收益。抓好工业扶贫和务工扶贫，加快天等县和大新县农民工创业园、龙州水口边贸加工扶贫产业园建设，吸引更多外出务工人员回乡创业，实现贫困群众在家门口就业；组织好贫困群众外出务工工作，增加工资性收入。

加快推进易地扶贫搬迁。抓好易地扶贫搬迁项目建设，力争年内实现搬迁1.2万人。做好易地扶贫搬迁后贫困户社会保障、后续产业发展等工作，确保贫困户搬得出、稳得住、能致富。

（七）坚持统筹城乡，加快建设山水园林城市。

加快提升中心城市品质。突出山水园林城市定位，做好崇左市城市总体规划修编工作，完善旧城区、水口湖片区、城西片区等控制性详细规划。加快中心城区7大系列31个重点项目的筹划和建设，推动友谊大道、花山路等路网提升改造工程开工建设，加快市体育中心、崇左大桥等项目建设，推动骆越大道、崇善大道、沿山路西段改造等项目实现竣工。积极抓好旧城改造，加快太平古城改造，推动丽江大桥、城西片区开发、左江两岸滨江路网及公园绿带建设等项目开工建设。拓宽城市建设筹资渠道，争取投入建设资金50亿元以上。力争中心城区建成区面积增加2平方公里。

提质扩容县城和特色城镇。修编完善各县（市、区）城市总体规划，加快扶绥、凭祥两个市级副中心城市建设，统筹推进大新、宁明、天等、龙州县域城镇化发展。继续抓好凭祥国家中小城市综合改革试点建设，加快凭祥-宁明-龙州一体化发展，推动扶绥空港-吴圩空港融合发展。大力实施中心镇、特色镇示范工程和书记工程，加快推进9个特色镇建设。力争全市城镇化率提高1.5个百分点以上。

推进美丽崇左乡村建设。提升清洁乡村、生态乡村建设成效，推进宜居乡村建设，抓好产业富民、服务惠民、基础便民三个专项活动。综合治理乡镇环境，推进43个乡镇片区垃圾处理中心和56个行政村垃圾处理终端建设，大力开展农村改厕改厨改圈工作。加强乡土村屯、传统村落和古树名木保护。

强化城市管理服务。大力开展自治区文明城市创建活动。深化城市管理体制改革，进一步理顺市本级与江州区城市建设管理职责，加强城市网格化管理。强化城市管理综合执法，深入开展“两违”综合整治，维护整洁有序的市容环境。

（八）坚持改善民生，推进社会事业全面发展。

加快发展教育。把发展教育作为完善城市功能、聚集城市人气、提升城市活力的重要抓手，年内在中心城区新建、改扩建3所幼儿园、4所小学，推动崇左高中扩建、广西大学附属中学崇左校区和崇左北大附属实验学校开工建设，打造崇左教育文化中心。深入实施教育“双百”工程，新建、迁建和改扩建一批学校。大力实施基础教育质量提升工程，确保天等县、大新县和宁明县通过国家义务教育均衡发展评估认定，扶绥县、江州区通过自治区义务教育均衡发展督导评估。积极推进南宁地区教育学院转制，加快推动广西东盟国际职业技术学院校区全面建设，大力支持广西城市职业学院、广西理工职业技术学院升本工作，力争广西城市职业学院崇左校区9月建成开学。加快老年大学建设。

扩大就业创业。落实就业创业政策，扎实开展创业培训，提升大众创业能力。统筹做好高校毕业生、城镇就业困难人员、退役军人、农村富余劳动力等各类群体就业工作，支持劳动者自主创业、自谋职业，确保全年城镇新增就业1.3万人、新增农村劳动力转移就业3万人，城镇登记失业率控制在4.2%以内。

加快完善社会保障体系。深入实施全民参保登记计划和机关事业单位养老保险制度改革，着力做好失地农民参加养老保险等工作。完善最低生活保障制度，推动社会保障制度由制度全覆盖实现人群全覆盖，进一步织密织牢社会化保障“安全网”。

大力发展文化体育事业。推进农家书屋、广播电视村村通、农村电影放映、公共文化场馆免费开放等工程，促进基本公共文化服务均等化。深入挖掘壮族文化底蕴，继续办好花山文化节，努力打造壮族“三月三”文化品牌。办好崇左市第三届运动会暨少数民族传统体育运动会。

着力提高人口质量和健康水平。进一步加强爱国卫生工作，确保成功创建自治区卫生城市。深入推进县级公立医院综合改革，启动城市公立医院综合改革，推进分级诊疗制度建设，巩固完善国家基本药物制度和基层运行新机制。推动市中医壮医医院整体搬迁项目建设，确保市儿童医院、妇幼保健院等项目实现竣工。坚持计划生育基本国策，依法组织实施“全面两孩”政策，促进人口均衡发展。大力推进“一园四基地”养老服务基础设施建设。

继续实施为民办实事工程。筹措资金 50亿元，全力抓好社保、健康、教育、水利、安居、农补、生态、文化、扶贫等为民办实事工程项目。

加强和创新社会治理。加快崇左市信用信息统一共享平台建设。创新社会治安综合治理，建立健全数据、指挥、行动一体化工作机制，年内全部建成市县乡村四级综治中心，力争全市重点公共区域视频监控系统覆盖率和联网率均达100%。依法惩治各类违法犯罪活动，进一步提高群众安全感。健全矛盾纠纷精准排查和精细化解机制。加快建立“一村（社区）一法律顾问”制度，打通基层公共法律服务“最后一公里”。加强应急管理体系建设，提高处置突发公共事件能力。强化食品药品监管，深入开展消防、安全生产隐患大检查，坚决遏制各类重大事故发生。

加强人才工作。深入实施人才强市战略，围绕做好“两篇大文章”、打好“四大攻坚战”培养发现和引进更多高层次人才。

加强民族宗教工作。深入开展民族团结进步创建活动，巩固民族团结良好局面。依法管理宗教事务，促进宗教和睦和谐。

支持国防和军队建设。做好国防教育、民兵预备役等工作，加强国防动员、国防后备力量建设和人民防空工作。深入开展双拥共建，推动军民深度融合发展。

三、 全面加强政府自身建设

全面贯彻落实党的十八届六中全会和自治区第十一次党代会精神，进一步增强政治意识、大局意识、核心意识、看齐意识，以全面从严治党统领政府各项工作，勤勉尽职，善谋善为，努力打造人民满意政府。

（一）强化宗旨意识，优化政府服务。坚持全心全意为人民服务的宗旨，强化用权为民、甘于奉献，为群众办实事、解难事，着力解决事关群众切身利益的突出问题。加快推进电子政务建设，推行“互联网+政务服务”，提高政府运行和服务效率。深入推进政务公开，强化各级政府网站建设和管理，推动决策、执行、管理、服务和结果“五公开”，提高政府透明度。做好重大政策解读和政务舆情回应工作，不断密切政群关系，提升政府公信力。

（二）强化依法行政，规范执政行为。坚持依法履职，把政府活动全面纳入法治轨道，自觉运用法治思维和法治方式推动工作，强化行政执法和监督。坚持重大行政决策集体讨论决定，提高行政决策科学化、民主化、法治化水平。自觉接受人大及其常委会监督和政协民主监督，切实做好人大代表建议和政协提案办理工作。广泛听取各民主党派、工商联、无党派人士和工青妇等人民团体意见。

（三）强化责任担当，狠抓工作落实。牢固树立政府工作“一盘棋”观念，强化政府部门之间、上下之间的主动协调、密切配合，形成齐抓共管的局面。推行“一线工作法”，推动干部在一线工作，任务在一线落实，问题在一线解决。强化落实意识，实行清单管理，加强问效问责，将市委、市政府的重大决策部署、重点工作任务层层量化分解，以目标倒逼责任，以时间倒逼进度，以督查倒逼质量，确保各项政策和任务不折不扣落到实处。

（四）强化廉洁自律，永葆清廉本色。巩固拓展“两学一做”学习教育成果，持之以恒转作风、正行风。深化党风廉政建设和反腐败斗争，落实“一岗双责”，严格执行廉洁自律准则，守住纪律处分条例规定的行为底线。健全权力运行制约和监督体系，着力整治侵害群众利益的不正之风和腐败问题，以作风建设新成效促进经济社会平稳健康发展。

各位代表！战略已经明确，目标已经确定。让我们紧密团结在以习近平同志为核心的党中央周围，在市委的坚强领导下，不负人民的重托，不辱群众的厚望，不忘初心，抢抓机遇，发力供给侧，打赢攻坚战，激发新动能，开拓新境界，为实现与全国全区同步全面建成小康社会而努力奋斗，以优异成绩迎接党的十九大胜利召开！
